# Mineral micronutrient status and spatial distribution among the Ethiopian population

**DOI:** 10.1017/S0007114522000319

**Published:** 2022-12-14

**Authors:** Adamu Belay, Dawd Gashu, Edward J. M. Joy, Murray R. Lark, Christopher Chagumaira, Dilnesaw Zerfu, Louise E. Ander, Scott D. Young, Elizabeth H. Bailey, Martin R. Broadley

**Affiliations:** 1 Center for Food Science and Nutrition, Addis Ababa University, Addis Ababa, Ethiopia; 2 Food Science and Nutrition Research Directorate, Ethiopian Public Health Institute, Gulele Sub City, Addis Ababa, Ethiopia; 3 Faculty of Epidemiology and Population Health, London School of Hygiene and Tropical Medicine, London, UK; 4 School of Biosciences, University of Nottingham, Sutton Bonington Campus, Loughborough, Leicestershire, UK; 5 Inorganic Geochemistry, Centre for Environmental Geochemistry, British Geological Survey, Nottingham, UK; 6 Rothamsted Research, West Common, Harpenden, Hertfordshire, UK

**Keywords:** Ethiopia, Geospatial prediction, Micronutrient surveys, Mineral micronutrients, Spatial variation, Variogram analysis

## Abstract

Multiple micronutrient deficiencies are widespread in Ethiopia. However, the distribution of Se and Zn deficiency risks has previously shown evidence of spatially dependent variability, warranting the need to explore this aspect for wider micronutrients. Here, blood serum concentrations for Ca, Mg, Co, Cu and Mo were measured (*n* 3102) on samples from the Ethiopian National Micronutrient Survey. Geostatistical modelling was used to test spatial variation of these micronutrients for women of reproductive age, who represent the largest demographic group surveyed (*n* 1290). Median serum concentrations were 8·6 mg dl^−1^ for Ca, 1·9 mg dl^−1^ for Mg, 0·4 µg l^−1^ for Co, 98·8 µg dl^−1^ for Cu and 0·2 µg dl^−1^ for Mo. The prevalence of Ca, Mg and Co deficiency was 41·6 %, 29·2 % and 15·9 %, respectively; Cu and Mo deficiency prevalence was 7·6 % and 0·3 %, respectively. A higher prevalence of Ca, Cu and Mo deficiency was observed in north western, Co deficiency in central and Mg deficiency in north eastern parts of Ethiopia. Serum Ca, Mg and Mo concentrations show spatial dependencies up to 140–500 km; however, there was no evidence of spatial correlations for serum Co and Cu concentrations. These new data indicate the scale of multiple mineral micronutrient deficiency in Ethiopia and the geographical differences in the prevalence of deficiencies suggesting the need to consider targeted responses during the planning of nutrition intervention programmes.

Multiple micronutrient deficiencies remain widespread globally with particular attention on Fe, folate, vitamin A, iodine and Zn through the analysis of biomarkers for population-level surveillance^(1)^. For many other micronutrients, there is limited knowledge of the prevalence of deficiencies based on biomarkers. This is likely due to the analytical challenges of measuring multiple micronutrients on small sample volumes and a lack of agreed thresholds/cut-offs and potential adjustments (e.g. for inflammation) for some micronutrients^(2,3)^. However, deficiencies of multiple micronutrients that are not routinely measured in national micronutrient surveys are likely to be widespread. For example, the deficiency risk of Ca based on food supply data is greater (54 %) than for Zn (40 %) in sub-Saharan Africa^(2,4)^. On the other hand, the deficiency risk for Cu is 1 % and less for Mg based on food supply data^(2)^.

Biomarker-based studies reporting mineral micronutrient status of the Ethiopian population except for Zn and Fe are limited. However, available reports suggest presence of multiple mineral micronutrient deficiency. A study analysing serum concentration of Mg, Zn, Se, Ca, Fe, Co and Mo shows the presence of high prevalence of Se (62 %) and Zn (47 %) deficiency among school age children (SAC) from Northwest Ethiopia. However, no or few children had Mg (2 %), Ca, Fe, Co, Mo and Cu/Zn deficiency^(5)^. In addition, a nationally representative study reported that 72 % of the Ethiopian population are Zn deficient^(6)^.

Nutrient deficiency is not always due to the lack of adequate intakes. Minerals may also antagonise the absorption and transportation of other minerals. Higher intake levels of one micronutrient in the diet or from supplements may competitively inhibit absorption of other micronutrients. This often occurs between nutrients possessing similar chemical characteristics as they can share similar receptors or transporting proteins^(7)^. In addition, deficiency of a micronutrient can affect the biological function of another micronutrient through disruption of the metabolic pathways. For example, Se deficiency can reduce the activity of the Se-dependant enzyme involved in the conversion of the biologically inactive thyroxine (T_4_) in to triiodothyronine (T_3_)^(8)^ indicating the need to concomitantly address deficiency of these two micronutrients. On the other hand, high dietary Ca concentration in the luminal environment reduces the rate of Mg absorption^(9)^, while Zn and Cu compete for absorption in the intestine^(10)^. Such available evidence of nutrient–nutrient interactions suggests that data on multiple micronutrient status of populations are important to understand not only the magnitude of the nutritional problem but also the context of the nutritional problem including presence of antagonistic or synergistic interactions to effectively design interventions.

The aim of this study was to determine the prevalence of multiple micronutrient deficiencies in Ethiopia and explore potential spatial dependencies of this variation based on each of the serum biomarkers. Prevalence of a deficiency is an important statistic to inform national policies, but spatial dependence is also significant. In the presence of spatial dependence, it may be expected that prevalence of deficiency changes from one location to another, and if spatial dependence is seen over sufficiently long distances, then this could imply that effective interventions differ between regions of a country, and that spatial information is needed to target them^(6,11,12)^. The focus is Ethiopia, where a National Micronutrient Survey (ENMS) was conducted in 2015. From the ENMS, the prevalence of Fe deficiency among young children (YC), SAC and women of reproductive age (WRA), as measured by ferritin and adjusted for inflammation, was 17·8 %, 9·1 % and 10·0 %, respectively^(13)^. Subsequently, multi-elemental analyses of serum samples were reported, both for Se^(11)^ and for Zn which included adjustments for inflammation^(6)^. These two studies provided strong additional evidence that Se and Zn deficiencies are widespread in Ethiopia, consistent with previous cross-sectional studies^(14)^. Furthermore, the prevalence of Se and Zn deficiency was spatially dependent, based on data for WRA, who represent the largest demographic group in the ENMS^(6,11)^. Thus, people living in some areas are likely to have a greater risk of micronutrient deficiency. For Se, spatial dependencies up to 200 km was reported^(11)^. Serum Se concentrations correlated positively with grain Se concentration, including a strong trend of increasing Se status from west to east Amhara linked to soil properties and landscape factors^(12)^. For Zn, spatial dependencies were found over shorter distances, of up to 45 km^(6)^. In the current study, the focus is on Ca, Mg, Co, Cu and Mo. This is because these elements could be reliably analysed in multi-element inductively coupled plasma-mass spectrometry and because there are accepted thresholds to indicate deficiency risks^(15)^. In addition, serum Zn concentration data reported previously^(6)^ were used to quantify Cu:Zn ratios, as a potential indicator for infection^(16,17)^.

## Materials and methods

### Study design and sample population

**Fig. 1.**
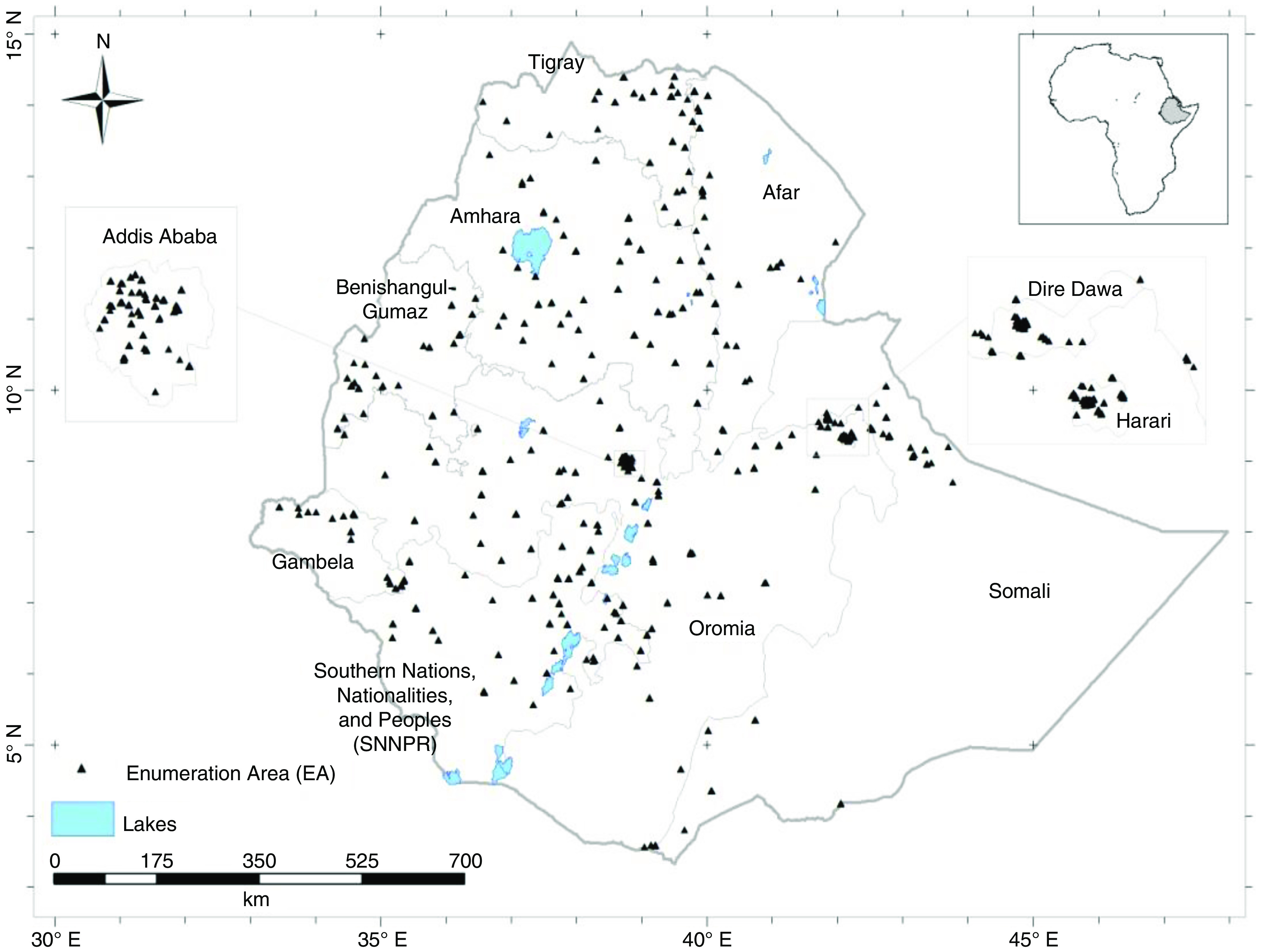
Locations of the centroids of *n* 346 Enumeration Areas from which study participants were recruited^(6,11)^.

The ENMS was designed to cover all regions and administrative cities (Addis Ababa and Dire Dawa) of Ethiopia (Fig. 1). The design of the ENMS is explained in detail elsewhere^(6,11,13,18)^. Briefly, the ENMS was a population-based, cross-sectional survey including young children (YC, aged 6–59 months, *n* 1100), SAC (aged 5–15 years, *n* 1500), non-pregnant (self-reported) (WRA, aged 15–49 years, *n* 1600) and men (aged 15–54 years, *n* 500) conducted between March and July 2015. The ENMS enumeration areas (EA), or clusters, are geographic areas deﬁned by the Central Statistics Agency for the Ethiopia Population and Housing Census^(19)^. For each region, the EA were selected based on standard probability proportional to size. EA contain on average 181 households (150 to 200)^(19)^. Within each selected EA, eleven households were randomly selected for enumeration.

This study used socio-demographic data collected from households in the ENMS, and newly generated data on micronutrients including Ca, Mg, Co, Cu and Mo concentrations measured in archived serum samples from the ENMS. The new micronutrient data were obtained only for those individuals from the ENMS survey for whom socio-demographic data were available, and where there was at least 0·3 ml of archived serum sample. Serum Zn concentration data were taken from Belay *et al*.^(6)^. A total of 3102 sample were included in the analysis.

### Ethics

This study was conducted according to the guidelines laid down in the Declaration of Helsinki, and all procedures involving human subjects were approved by the National Research Ethics Review Committee at the Ministry of Science and Technology, Ethiopia (Reference 3.10/433/06). Written informed consent and assent were obtained for adult and child participants, respectively. This study was also approved by the Research Ethical Review Committee at the Ethiopian Public Health Institute (Protocol EPHI-IRB-140-2018). Archived serum samples were transferred from storage at EPHI to the University of Nottingham, UK for analysis under a Material Transfer Agreement.

### Data collection and analysis

#### Socio-demographic data

The ENMS collected socio-demographic information using pre-tested questionnaires. The data collectors were trained for three weeks on data collection and quality control following pilot testing in a cluster which was not included in the final ENMS. Questionnaires were refined after the pilot testing and before the survey data collection began.

#### Collection, processing and analysis of serum micronutrients

Detail information about blood collection has been described previously^(11,13,18)^. Briefly, trained phlebotomist collected blood samples from the antecubital veins of participants following WHO blood collection guidelines^(20)^. Blood and serum samples were aliquoted for a range of analyses^(13)^. Backup sample aliquots were kept at -80 °C to enable analytical cross-checks and additional analyses such as those reported previously^(6,11)^ and in this study.

#### Serum micronutrient analysis

Elemental concentrations of Ca, Mg, Co, Cu and Mo in serum samples were determined using inductively coupled plasma-mass spectrometry (Thermo Fisher Scientific iCAPQ, Thermo Fisher Scientific) as reported previously^(6,11)^. Samples were introduced, via a single line, from an autosampler incorporating an ASXpress™ rapid uptake module (Cetac ASX-520, Teledyne Technologies Inc.) through a perfluoroalkoxy (PFA) Microflow PFA-ST nebulizer (Thermo Fisher Scientific). All samples and external multi-element calibration standards were diluted as 0·3 ml added to 6 ml of a solution containing: (i) 0·5 % HNO3 (Primar Plus grade); (ii) 2·0 % methanol (Fisher Scientific UK Ltd); and (iii) three internal standards including ^72^Ge (10 µg/l), ^103^Rh (5 µg/l), ^193^Ir (5 µg/l) (SPEX Certiprep Inc.). Calibration standards included: (i) Co, Cu and Mo (0, 20, 40, 100 µg/l; Claritas-PPT grade CLMS-2; SPEX Certiprep Inc.); and (2) Ca and Mg (0, 10, 20, 30 mg/l; PlasmaCAL, SCP Science). The inductively coupled plasma-mass spectrometry was operated in ‘collision-reaction cell mode’, with kinetic energy discrimination, using He for all elements. The quadrupole dwell time was 0·1 s and 150 scans were used to obtain an average measurement of signal intensity (CPS; counts-per-second).

The limit of detection for all elements was measured as 3 × standard deviation of 10 operational blanks; the limits of detection of Ca, Mg, Co, Cu and Mo were 91·3, 3·12, 0·013, 0·407 and 0·064 µg l^−1^, respectively. The limit of quantification (LOQ) was calculated as 10 × standard deviation. Values of LOQ (µg l^−1^) were 304 for Ca, 10·4 for Mg, 0·044 for Co, 1·36 for Cu and 0·212 for Mo.

Accuracy was verified by the use of two appropriate certified reference materials (CRM) (Seronorm™ L-1 (Lot 1801802) and Seronorm™ L-2 (Lot 1801803)); Nycomed Pharma AS); these were typically run at the start and the end of sample batch runs and they were prepared identically to samples and calibration standards. A total of 24 individual CRM analyses were undertaken for both L-1 and L-2. The average recoveries were recorded for Seronorm™ L-1 and Seronorm™ L-2. The average recoveries (%) were 89 for Ca, 100 for Mg, 100 for Co, 88 for Cu and 113 for Mo in Seronorm^TM^ L-1 CRM and in Seronorm^TM^ L-2 CRM, the average recoveries (%) were 92 for Ca, 102 for Mg, 101 for Co, 85 for Cu and 95 for Mo. All micronutrients in CRM were accredited except for Mo.

#### Data analysis

From the total of 3373 samples, 271 observations were excluded due to missing GPS coordinates, missing information on time of food intake during data collection, or a lack of socio-demographic data and analytical outlier datum (3xIQR)^(21)^. Thus, 3102 observations were included for descriptive statistical analyses of the prevalence of micronutrient deficiencies using STATA (Version 14.0, StataCorp., LLP). Prevalence of Zn deficiency among all demographic groups in Ethiopia was previously determined^(6)^ as suggested by King *et al*.^(22)^. Cut-off values for Ca, Mg, Co, Cu and Mo deficiency were defined at their serum levels of 8·4 mg/dl, 1·8 mg/dl, 0·22 μg l^−1^, 75 μg/dl and 0·02 μg/dl, respectively, for all demographic groups^(15)^. Ratios of Ca:Mg^(9)^ and Cu:Zn^(16)^ were calculated as indicators for chronic disease and presence of inflammation in the population, respectively. Pearson’s correlation coefficients were estimated for exploration of association between pairs of elements.

#### Spatial determination of micronutrient deficiencies

Geostatistical modelling was undertaken to reveal the spatial dependence of micronutrient deficiencies, using methods described previously^(6,11,23)^, and more detail on the analytical methods is presented in the Supplementary Information S1. Briefly, ordinary kriging was used to predict serum concentrations at unsampled location by interpolation from the measured data. In ordinary kriging, the interpolation is a weighted linear combination of the observations, with weights computed to minimise the expected squared prediction error (kriging variance). This measure of uncertainty of the predictions was calculated (Supplementary Information Table S1). The ordinary kriging prediction is the mean of the prediction distribution at the target location, and the kriging variance is the variance of the prediction distribution. Ordinary kriging predictions were computed on the nodes of a 60-metre square grid. These predictions, and the kriging variances, were then presented as a map using ArcGIS (10.4.1). For all micronutrients, the spatial analysis is focused on WRA because there were greater numbers of WRA and cover a large number of EA (320 out of a total of 346 EA). The statistical summary for serum micronutrients of WRA is shown in Supplementary Information Fig. S1.

## Results

### Population demographic and micronutrient data

This study included 474 YC, 935 SAC, 403 men and 1290 WRA, with highest participants from Oromia region and lowest number of participants from Dire Dawa Administrative city. The mean age of YC, SAC, men and WRA was 3·1 ± 1·0, 9·6 ± 2·7, 30·1 ± 10·5 and 28·2 ± 8·9 year, respectively. The detailed demographic information is reported elsewhere^(6,11)^.

The median serum concentration of micronutrients in the study groups is shown in Table 1. Median serum concentrations of Ca, Mg, Co, Cu and Mo were 8·6 mg/dl, 1·9 mg/dl, 0·4 µg l^−1^, 98·8 µg/dl and 0·2 µg/dl, respectively, and ratios of Ca:Mg and Cu:Zn were 4·4 and 1·8, respectively. The median serum concentrations of micronutrients differed between the regions, with the highest serum Ca and Mg concentrations observed in Dire Dawa followed by Amhara. Serum Ca:Mg ratio was high (>4) in all regions. Serum Co concentration was highest in Benishangul-Gumuz and lowest in Addis Ababa. Serum Cu concentration was highest in Gambela and lowest in SNNP. Younger children had lower serum concentrations of all micronutrients except Cu compared with their counterparts. Young children had the largest serum Cu:Zn ratio. Urban residents had larger serum micronutrient concentrations than rural residents, except for Cu. There were little differences in median micronutrient status between groups based on the educational status of the head of household.

**Table 1.** Median (Q1, Q3) serum concentrations of micronutrients among the Ethiopian population, according to region, demographic group, location of residence and educational status (Mean and quartiles)

		Ca, mg dl^−1^ *		Mg, mg dl^−1^ *		Ca:Mg ratio*		Co, µg l^−1^ *		Cu, µg dl^−1^ *		Zn, µg dl^−1^ *		Cu:Zn ratio*		Mo, µg dl^−1^ *	
Variable	*n*	Median	Quartiles	Median	Quartiles	Median	Quartiles	Median	Quartiles	Median	Quartiles	Median	Quartiles	Median	Quartiles	Median	Quartiles
Region																	
Addis Ababa	184	8·5	8·2, 8·8	2·0	1·9, 2·1	4·3	4·0, 4·5	0·26	0·18, 0·41	110·5	94·7, 1·24·4	67·9	57·9, 72·5	1·8	1·5, 1·9	0·17	0·12, 0·25
Afar	266	8·5	7·9, 8·9	1·9	1·8, 2·0	4·5	4·2, 4·6	0·36	0·22, 0·54	102·4	89·9, 118·6	58·6	50·7, 65·9	1·8	1·5, 2·2	0·19	0·14, 0·27
Amhara	480	9·0	8·5, 9·3	2·0	1·9, 2·2	4·4	4·1, 4·7	0·52	0·36, 0·75	101·4	90·3, 113·2	57·4	49·9, 64·4	1·8	1·5, 2·1	0·11	0·07, 0·17
Benishangul-Gumuz	211	8·3	7·9, 8·7	1·9	1·8, 2·0	4·4	4·1, 4·7	0·59	0·38, 0·95	96·6	83·5, 112.·2	57·6	49·9, 65·1	1·7	1·4, 2·0	0·14	0·10, 0·21
Dire Dawa	156	9·2	8·8, 9·6	2·0	1·9, 2·2	4·5	4·2, 4·7	0·39	0·18, 0·60	99·2	87·7, 118 7	60·8	55·4, 67·4	1·7	1·4, 1·9	0·19	0·14, 0·26
Gambela	192	8·4	7·9, 8·9	1·9	1·8, 2·1	4·3	4·1, 4·5	0·42	0·29, 0·68	115·2	99·4, 128·3	58·3	52·1, 65·5	1·9	1·6, 2·3	0·20	0·14, 0·30
Harari	167	8·8	7·8, 9·2	1·9	1·8, 2·1	4·5	4·2, 4·7	0·39	0·24, 0·64	98·1	84·1, 116·6	55·7	46·6, 63·7	1·9	1·6, 2·2	0·19	0·15, 0·28
Oromia	515	8·4	7·8, 8·8	1·9	1·7, 2·0	4·4	4·2, 4·7	0·45	0·28, 0·81	96·9	85·2, 109.·5	54·5	46·3, 62·2	1·8	1·5, 2·2	0·15	0·10, 0·23
SNNP	390	8·4	7·9, 8·8	1·9	1·7, 2·0	4·4	4·2, 4·7	0·32	0·22, 0·56	96·2	84·6, 110·1	57·3	49·4, 63·5	1·7	1·4, 2·1	0·23	0·16, 0·34
Somali	201	8·7	8·3, 9·1	1·9	1·8, 2·1	4·5	4·2, 4·8	0·47	0·22, 0·78	110·6	99·2, 127·7	59·7	53·2, 67·1	1·9	1·6, 2·2	0·16	0·12, 0·22
Tigray	340	8·3	7·7, 8·6	1·9	1·8, 2·0	4·3	4·1, 4·5	0·42	0·28, 0·63	97·4	86·6, 111·9	54·0	46·7, 60·8	1·9	1·5, 2·2	0·18	0·13, 0·25
Total	3102	8·6	8·0, 9·0	1·9	1·8, 2·1	4·4	4·2, 4·7	0·43	0·27, 0·73	98·8	87·3, 112·7	56·2	48·6, 63·5	1·8	1·5, 2·1	0·16	0·10, 0·24
Demographic group																	
YC	474	7·3	6·9, 7·7	1·7	1·6, 1·8	4·4	4·1, 4·6	0·43	0·30, 0·69	101·8	89·1, 116·1	46·1	40·4, 53·3	2·2	1·9, 2·6	0·15	0·11, 0·22
SAC	935	8·6	8·1, 9·9	1·9	1·8, 2·1	4·4	4·2, 4·6	0·45	0·31, 0·71	100·9	89·5, 113·0	55·9	48·9, 61·9	1·8	1·6, 2·1	0·16	0·11, 0·26
Men	403	8·8	8·4, 9·1	1·9	1·8, 2·0	4·5	4·3, 4·8	0·39	0·25, 0·67	91·7	83·2, 100·4	61·1	52·6, 67·3	1·5	1·3, 1·8	0·15	0·10, 0·22
WRA	1290	8·7	8·3, 9·2	1·9	1·8, 2·1	4·5	4·2 ,4·7	0·43	0·25, 0·76	99·7	87·3, 113·3	59·6	51·4, 65·7	1·7	1·4, 2·0	0·16	0·10, 0·24
Residence																	
Rural	2289	8·5	7·9, 9·0	1·9	1·8, 2·1	4·4	4·2, 4·7	0·45	0·29, 0·76	98·7	86·9, 111·9	55·4	47·6, 62·5	1·8	1·5, 2·2	0·16	0·10,0·24
Urban	813	8·7	8·3, 9·1	2·0	1·8,2·1	4·4	4·2, 4·7	0·35	0·22, 0·55	101·1	88·8, 115·6	61·7	54·9, 67·5	1·7	1·4, 2·0	0·15	0·10,0·22
Educational status																	
Literate	1548	8·5	8·1, 9·0	1·9	1·8, 2·1	4·4	4·2, 4·7	0·41	0·26, 0·69	98·4	86·6, 112 6	57·6	49·6, 64·7	1·7	1·5, 2·1	0·16	0·10, 0·24
Illiterate	1554	8·6	7·9, 9·0	1·9	1·8, 2·1	4·4	4·2, 4·7	0·47	0·29, 0·75	99·5	87·6, 112·8	55·4	47·7, 62·3	1·8	1·5, 2·2	0·16	0·10, 0·24

SNNP, Southern nations, nationalities and peoples; YC, young children; SAC, school age children; WRA, women of reproductive age.

* Values in brackets are Q1:25th percentile and Q3:75th percentile.

Of the total study population serum samples 41·6 %, 29·2 %, 15·9 %, 7·6 % and 0·3 % were Ca, Mg, Co, Cu and Mo deficient, respectively (Table 2). Among the study groups, 34·9 % of the population was deficient in at least one of the micronutrients, and 22·4 % and 20·7 % of study population showed two and three coexisting micronutrients deficiencies, respectively. Among demographic groups, a high micronutrient deficiency burden was observed in young children, except for Mo.

**Table 2.** Micronutrient deficiency prevalence (%) among the Ethiopian population, according to region, demographic group, location of residence and educational status

Variable	*n*	Ca	Mg	Co	Cu	Zn	Mo
Region							
Addis Ababa	184	39·1	15·1	36·4	5·3	27·2	0·00
Afar	266	40·3	27·7	23·5	8·9	63·2	0·00
Amhara	480	21·1	17·1	6·5	2·9	74·1	1·14
Benishangul-Gumuz	211	54·7	28·7	9·4	12·9	69·9	0·00
Dire Dawa	156	9·2	5·5	33·2	7·5	66·4	0·00
Gambela	192	49·5	20·3	11·5	2·7	64·4	0·00
Harari	167	37·5	27·6	20·5	10·6	71·8	0·00
Oromia	515	51·9	37·2	16·0	8·5	80·0	0·00
SNNP	390	46·7	33·6	25·8	11·5	73·2	0·00
Somali	201	30·4	23·3	24·4	2·6	66·5	0·43
Tigray	340	59·5	29·5	12·5	5·8	78·4	0·00
Total	3102	41·6	29·2	15·9	7·6	75·1	0·33
Demographic group							
YC	474	90·5	76·1	12·1	7·9	92·1	0·00
SAC	935	38·2	20·9	11·2	7·6	75·8	0·95
Men	403	23·1	23·4	17·7	7·7	73·9	0·00
WRA	1290	32·2	20·5	20·7	6·3	67·2	0·04
Residence							
Rural	2289	42·9	30·7	14·2	7·6	78·0	0·43
Urban	813	33·9	20·4	25·5	4·5	58·1	0·00
Household head educational status							
Educated	1548	40·5	29·5	18·7	7·2	72·5	0·00
Non-educated	1554	42·6	28·9	13·6	7·1	77·3	0·61

SNNP, Southern nations, nationalities and peoples; YC, young children; SAC, school age children; and WRA, women of reproductive age.

The correlation coefficients indicated the presence of association between pairs of micronutrients, namely Ca-Mg (*r* = 0·70), Cu-Mg (*r* = 0·26), Zn-Mg (*r* = 0·31), Ca-Zn (*r* = 0·42) and Cu-Zn (*r* = 0·12) as indicated in Table 3.

**Table 3.** Pearson’s correlation coefficients between serum micronutrients of the Ethiopian population

Micronutrient	Mg	Ca	Co	Cu	Zn	Mo
Mg	1·00					
Ca	0·70	1·00				
Co	0·00	0·01	1·00			
Cu	0·26	0·21	0·02	1·00		
Zn	0·31	0·42	0·03	0·12	1·00	
Mo	0·04	0·00	0·01	0·01	0·04	1·00

### Spatial analysis of women of reproductive age serum micronutrient data

The WRA comprised the largest demographic group within the analysed samples, so these were used to explore whether spatial variation exists within the survey data. Variogram values were estimated with the alternative estimators and plotted against lag distance, along with models fitted by weighted least squares using R software. If the exponential variogram model for estimates by a particular estimator had a smaller value of AIC than the corresponding pure nugget model, then this exponential model was subject to cross-validation. A model (from one of the estimators) was selected by reference to the value of the median standardised squared prediction error. There was no evidence to support a model of spatial dependence for the data on Co, Cu and the Cu:Zn ratio. However, the AIC for the spatially dependent model was smaller than for the pure nugget model in the case of Ca, Mg, Mo and the Ca:Mg ratio. Furthermore, for all these variables, the model fitted to the estimates by Matheron’s estimator was selected as the median SSPE for the cross validation of this model fell within the 95 % confidence interval for this statistic under a valid model in all cases. This indicates that there is no basis for mapping spatial variation in the Co, Cu and Cu:Zn serum data, and these data are not further presented in this section. In contrast, Ca, Mg, Mo and the Ca:Mg ratio showed spatial dependence at distances from 140 to 500 km, with Ca:Mg having the shortest range of spatial dependence, at which the variogram is approximately flat (150 km) and Ca showing spatial dependence over the longest distances (over 500 km).

**Fig. 2.**
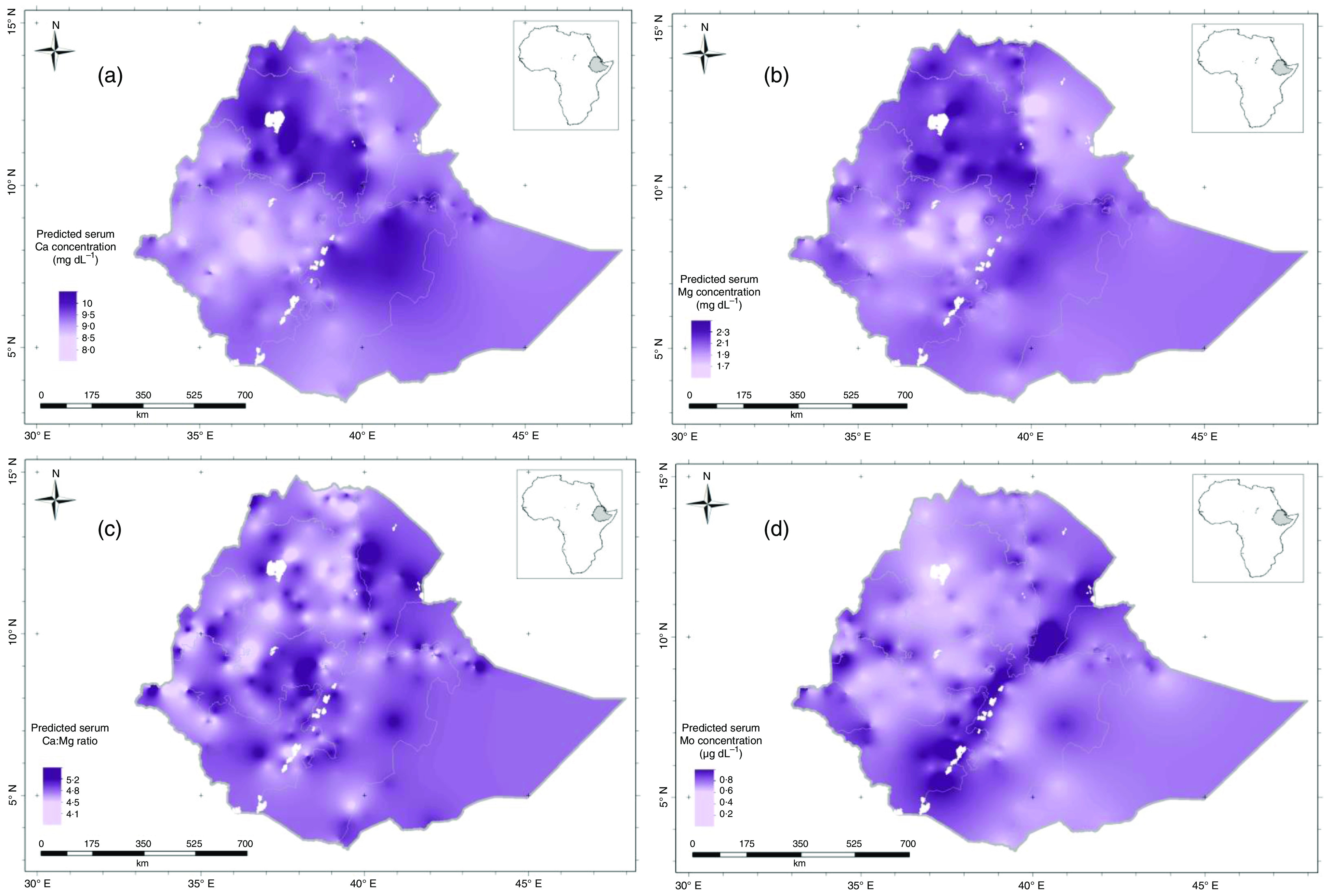
Predicted serum: (a) Ca; (b) Mg; (c) Ca:Mg ratio and (d) Mo concentration (the mean of the prediction distribution) for women of reproductive age in Ethiopia.

The kriging interpolation maps (Fig. 2(a)–(d)) show higher serum Ca and Mg concentrations in north western and south eastern areas, including large parts of Amhara, Dire Dawa and eastern Oromia regions, shaded in deep purple colours. Lower concentrations of these elements were predicted in south western, north eastern areas and along the Rift Valley, including parts of Tigray, Benishangul-Gumuz, Gambella and western and southern parts of Oromia regions, shaded in light purple colours. The highest values of serum Ca:Mg ratio were observed in north eastern, south western, south eastern and central parts of Ethiopia, appearing as disconnected patches of deep purple colours. Higher serum Mo concentrations can be seen in the Rift Valley area. The lower serum Mo concentrations were observed in north western and south eastern parts of the country including Amhara, Benishangul-Gumuz, east and south Oromia regions.

**Fig. 3.**
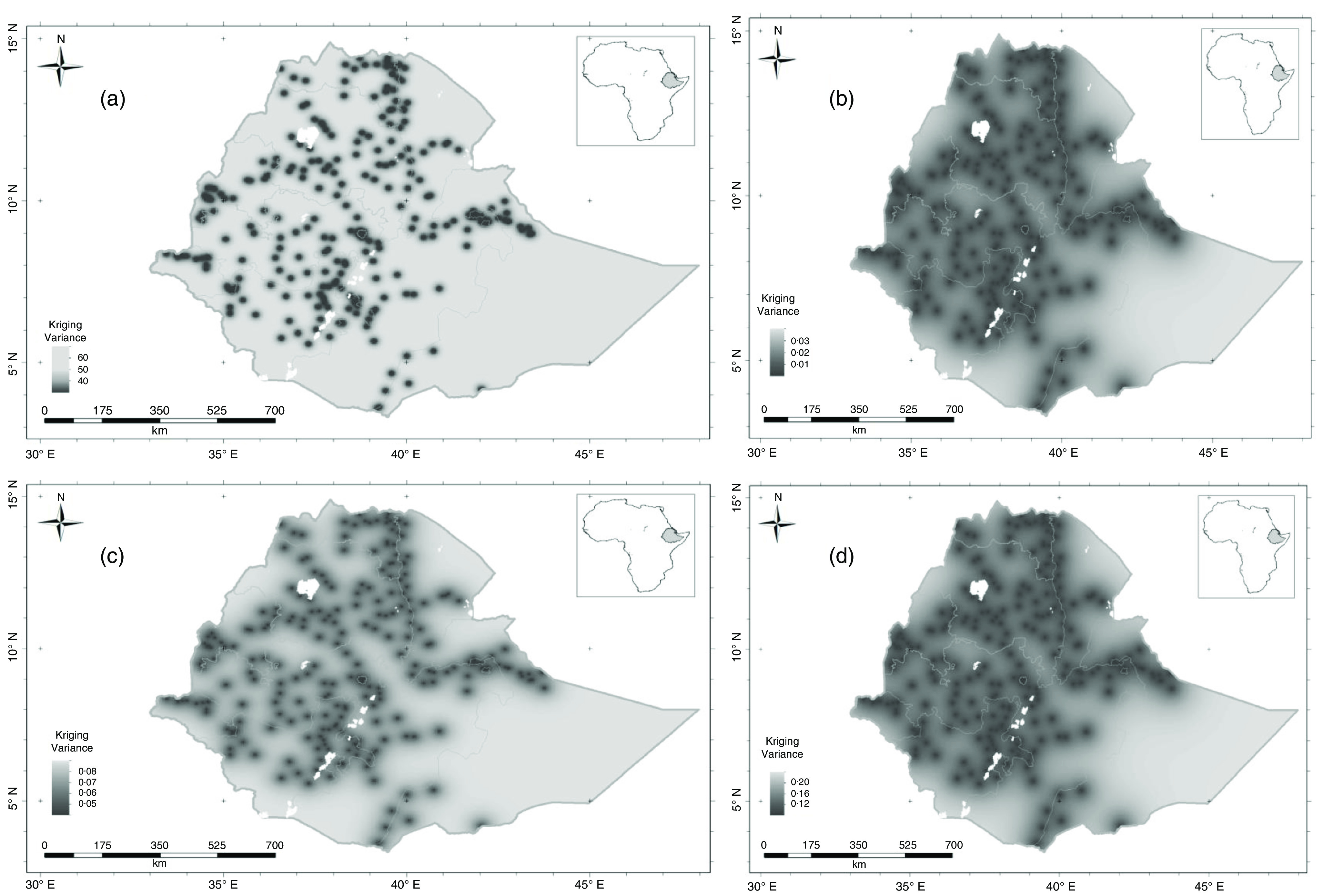
Serum micronutrient kriging variance for women of reproductive age in Ethiopia: (a) Ca; (b) Mg; (c) Ca:Mg ratio and (d) Mo.

The kriging variance of serum Ca, Mg, Ca:Mg ratio and Mo concentrations for WRA are shown in Fig. 3(a)–(d); these are the expected kriging variance computed based on the variogram model. These are the measures of prediction uncertainty that is minimised by ordinary kriging. Interpolation error is expected because of the spatial variation of our target variables. These kriging variances allow us to visualise the uncertainties and avoid arbitrary decisions as to where spatial prediction is reliable from a particular distribution of observations. For all micronutrients, there is greater uncertainty in some parts of the country, shaded in light grey, because of the sparse distribution of observations^(24)^. In these circumstances, the predicted value tends to the mean of the observations, and the kriging variance becomes large (light grey areas). If decisions on nutrition intervention programs are needed where the kriging variance is large, then local sampling would be essential.

**Fig. 4.**
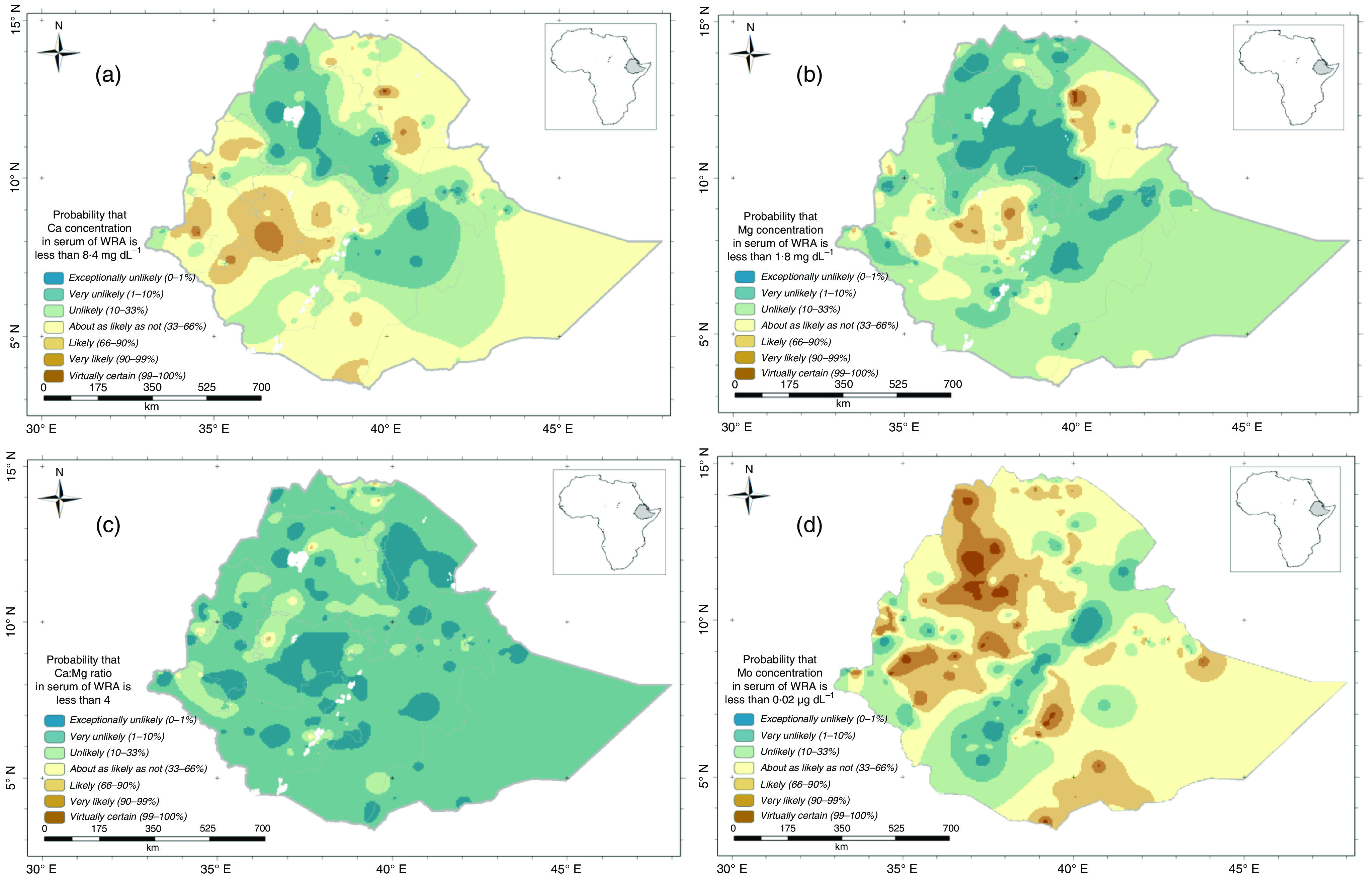
Probability that serum micronutrient concentration of women of reproductive age would fall below threshold for serum: (a) Ca; (b) Mg; (c) Ca:Mg and (d) Mo.

The combined predicted concentration and uncertainty estimations outputs from the geostatistical analysis allow us to visualise occurrence that the probability of both risk micronutrient deficiency or that there are high micronutrients concentration at a national scale. Fig. 4(a)–(d) shows the probability that the mean serum Ca, Mg, Ca:Mg ratio and Mo concentration of WRA in an EA is below a threshold of 8·4 mg/dl, 1·8 mg/dl, 4 and 0·02 μg/dl, respectively. Because continuous probabilities are not always easily interpreted by diverse stakeholders, we also present these probabilities using the ‘calibrated phrases’ of the Intergovernmental Panel for Climate Change^(25)^, which are designed for the communication of uncertain information to data users unfamiliar with probability. This approach has been used elsewhere^(11,23,26)^.

From the Ca and Mg probability maps (Fig. 4(a)–(d)), we can see that in the north west (Amhara region) and east of Oromia and Dire Dawa regions, WRA are ‘unlikely’ through to ‘exceptionally unlikely’ to be Ca and Mg deficient. However, the likelihood of Ca and Mg deficiencies are generally greater in some north eastern and western areas of Ethiopia, which may increase the risk of some diseases like pre-eclampsia and hypomagnesemia, respectively. In addition, from the Ca:Mg ratio probability map, we observed that WRA in most parts of the country are ‘unlikely’ through ‘exceptionally unlikely’ to have a low Ca:Mg ratio (<4). Beyond the Rift Valley areas, WRA are ‘likely’ to ‘virtually certain’ to have low serum Mo concentrations.

## Discussion

This study revealed a high prevalence of deficiencies of Ca, Mg and Co but low prevalence of deficiencies of Cu and Mo in Ethiopia. The prevalence of micronutrient deficiencies varied spatially for Ca, Mg and Mo with a high burden of deficiency observed in young children. A greater prevalence of micronutrients deficiencies was observed among those living in rural areas, except for Co.

In the previous study based on food supplies data, the mean risk of Ca and Mg deficiencies for Eastern parts of Africa was 69 % and 1·2 %, respectively^(2)^. Furthermore, the Ethiopian food consumption survey result revealed that the mean Ca intake for women was 501 mg/d at the national level which is far less than the cut-off (900 mg/d) defined for a low intake indicating that about 88 % of the population are at risk of Ca deficiency^(27)^. In the present study, 41 % and 29 % of the study population were deficient in Ca and Mg, respectively, based on serum concentration thresholds. However, it is known that biomarker data of micronutrients are more reliable compared with estimation of deficiency risk using food supply data. Young children had the highest prevalence of Ca (90 %) and Mg (76·1 %) deficiency, although these are based on adult thresholds. Countries with low dietary intake of dairy products have low Ca status^(28)^. In Ethiopia, milk and dairy product consumption is relatively low^(27)^. Rural residents were more likely to be below the thresholds for serum Ca and Mg concentrations than urban residents, which may reflect the intake of flesh, dairy product and eggs which are good source of these minerals are higher in urban than in rural areas^(29)^. Serum Ca and Mg show strong positive association (*r* = 0·70).

Ca and Mg are essential for bone formation and for numerous kinds of metabolisms in human body^(30)^. They are intimately related to each other and have been reported to collectively influence metabolic functions such as hypertension and insulin resistance^(31,32)^. A study in a Chinese population revealed that individuals with metabolic syndrome had higher whole blood levels of Mg but lower Ca compared with healthy controls^(30)^. A study in Nigeria also shows that pre-eclampsia prevalence was higher in women with a Ca:Mg ratio greater than 2^(33)^. In our study, the mean and median Ca:Mg ratio was > 4, which may increase the risk of developing chronic diseases including hypertension, cardio-vascular and diabetes^(34,35)^.

Cu and Zn are involved in numerous aspects of cellular metabolism because they allow many critical enzymes to function properly^(10)^. Cu deficiency is relatively low among all of the demographic groups in Ethiopia based on this threshold and consistent with food supply data^(2)^. In contrast, Zn deficiency is widespread in the country^(6)^, with YC more likely to be Zn deficient than SC, men and WRA. This observation agrees with Ethiopian Food Consumption survey that reported 95 % of YC were susceptible for Zn deficiency based on food intake^(36)^.

An elevated serum Cu:Zn ratio indicates the presence of inflammation in addition to C-reactive protein and *α*1-acid glycoprotein biomarkers. This is because inflammation and infection can reduce serum Zn during the acute phase response due to the redistribution of the serum Zn into liver and other tissues. Furthermore, acute infection leads to increased serum Cu concentration^(16)^. Both responses cause serum Cu:Zn ratio to increase. In a previous study on SAC in Ethiopia, the Cu:Zn ratio was greater than 2 indicating a high prevalence of infection^(5)^. Similarly, in the present study, the Cu:Zn ratio of YC was >2, which is consistently indicating the presence of a high burden of infection in young children^(37)^.

Co is a key component of cobalamin (vitamin B_12_) and is required to produce haemoglobin and erythrocytes^(38)^. It also plays important roles in the formation of amino acid and neurotransmitters ^(39)^. The median concentration of serum Co in Ethiopia was 0·4 mg l^−1^. There was no difference among different demographic groups and their residential area. We observed that 15·9 % of the study population had Co deficiency with highest prevalence in Addis Ababa and Dire Dawa administrative areas. Among demographic groups, the highest prevalence of Co deficiency was in WRA (20·7 %). The ENMS reported that 15·1 % of the surveyed WRA were deficient in vitamin B_12_ with the highest prevalence observed in Dire Dawa (39·2 %, *n* 1374)^(13)^. This Co deficiency in women might be associated with disturbance in vitamin B_12_ synthesis, as a result it might trigger anaemia^(40)^.

Mo is an important component of enzymes such as xanthine dehydrogenase, aldehyde oxidase and sulphite oxidase^(41,42)^. The median concentration of serum Mo in Ethiopia was 0·2 μg dl^−1^ within the range of 0·01–2·1 μg dl^−1^. This is high compared with other countries like Japan, Belgium, USA, UK, Sweden, Hungary, Germany and Zaire that serum Mo concentration is in the range of 0·12–0·22 μg dl^−1(42)^. Similar to the distribution of Co, there was no significant difference in serum Mo status among demographic groups and their residential area. However, there were spatial differences between regions, with Mo deficiency prevalence was 1·1 % in Amhara and 0·4 % in the Somali region. Food groups with high Mo concentrations are plant foods such as cereals, pulses, nuts and their product^(41,42)^. The national food consumption survey reported that most of the food sourced in Ethiopia are plant based^(36)^.

Mineral micronutrients of similar properties share same transporting and receptor proteins hence exhibit interaction at the site of absorption or during metabolic processes. For example, Ca and Mg interact and a change in Ca may affect Mg balance and vice versa^(43)^. Similarly, in the present study, there was an association between the mineral micronutrients. The present study has a hierarchical sampling hence the variation comprises contributions from variations among individuals within households, from households within enumeration areas and from enumeration areas. In addition, the variation varies between scales, and correlations will very likely do the same. The relative contribution of the different sources of variation to mineral concentrations is determined entirely by the sampling decisions (number of households and enumeration areas), and so these correlations do not only reflect the actual processes at the different scales, but those decisions too. As a result, these correlations can only be exploratory and should be interpreted with caution. The significance levels are very questionable, thus are not included.

Regarding the geostatistical output, the variogram model shows spatial variability of serum Ca concentration, followed by Mg, Mo and Ca:Mg ratio. Several factors may contribute to variations in serum micronutrients concentrations. These include physiological status including presence of infection and influences including dietary patterns, age and sex^(44,45)^. In addition, in Ethiopia, spatial factors linked to soil types and landscape features appear to be strong drivers of longer range variation in micronutrients status^(2,14,23)^. Food systems are highly localised in Ethiopia, particularly in rural areas, with a large proportion of dietary intakes met through subsistence production or purchases of locally produced food^(46)^. Thus, individuals’ status reflects the soil types and landscapes where they reside. While the variogram model represents no spatial structure for serum Cu, Cu:Zn ratio and Co concentrations (online Supplementary Table S1).

The information produced in this study can be used for a baseline assessment for further studies, but it can also help to target the most affected areas for appropriate food-based interventions. The information used to apply further sampling requirements may need if the kriging variance is very large as seen in eastern part of the country where the sampling data are spares.

The main study strengths include (i) the analysis of different micronutrient concentrations in the serum of different demographic groups; (ii) the large sample size; (iii) the use of inductively coupled plasma-mass spectrometry by which multiple elements can be reliably analysed simultaneously and with high sensitivity and (iv) the application of geostatistical models to predict the unsampled area with the probability map and visualise the uncertainty of the prediction. However, the present study lacks detailed information on socio-economic status, data on dietary intake and soil and crops data at national level. But, soil and crop data in Amhara regions show a strong correlation Gashu *et al*.^(12)^. These data may help us to explore true determinants of micronutrient status and deficiency in the population studied. We apply the same cut-off for all demographic groups except for Zn may increases high deficiency rate in children. In addition, serum Ca concentration is not a reliable marker of Ca status as it is subjected to strong homoeostatic regulation and changes only after chronic Ca deprivation^(47,48)^; hence, the Ca result of the present should be interpreted in caution.

### Conclusion

This is the first national study to demonstrate the serum micronutrient concentration in Ethiopia. There is a high prevalence of Ca, Mg and Co deficiencies, with YC likely to be most affected with micronutrient deficiency. However, there is a need to establish deficiency thresholds for all demographic groups to gain a better estimation of micronutrient deficiency prevalence, as recommended by Ewers *et al*.^(49)^. The variogram models show that Ca, Mg, Ca:Mg ratio and Mo have spatial dependence in the distance range 140–500 km, whereas Co, Cu and Cu:Zn ratio shown spatially independent. These data contribute on the serum micronutrients status of Ethiopian population and can be helpful in the subsequent studies as baseline as well as for the cost-effective intervention programme to mitigate micronutrients deficiency in high burden area.

## Supplementary material

For supplementary material accompanying this paper visit https://doi.org/10.1017/S0007114522000319. click here to view supplementary material
